# Pioneering psycho-oncology: A collaborative journey for medical and psychology students at *e*cancer-Tata Medical Center Kolkata Psycho-oncology Congress 2024

**DOI:** 10.3332/ecancer.2025.1828

**Published:** 2025-01-17

**Authors:** Soumitra Shankar Datta, Heena Sheth, Sharmili Ghosh, Srijan Das, Jigeesha Ghosh, Dishari Choudhury, Arnab Mukherjee, Soumita Ghose, Romy Biswas, Maria Castrillo Gil, Carlos Andres Gamboa Alfaro, Mary Guevara, Danny Burke, Sujit Sarkhel, Jai Ranjan Ram

**Affiliations:** 1 Department of Palliative Care & Psycho-oncology, Tata Medical Center, New Town, Rajarhat, Kolkata 700160, India; 2 Institute of Clinical Trials and Methodology, University College London, 90 High Holborn, London WC1V 6LJ, UK; 3 Department of Psychiatry, Central Institute of Psychiatry, Kanke, Ranchi 834006, India; 4 Department of Administration and Policy, Tata Medical Center, Major Arterial Road, New Town, Rajarhat, Kolkata 700160, India; 5 Department of Community Medicine, Maharaja Jitendra Narayan Medical College, and Hospital, Vivekananda Street, Pilkhana, Cooch Behar 736101, West Bengal, India; 6 *e*cancer, 13 King Square Avenue, Bristol BS2 8HU, UK; 7 Department of Psychiatry, IPGMER and SSKM Hospital, 7, DL Khan Road, Kolkata 700025, West Bengal, India; 8 Mental Health Foundation, 6, Andul Raj Road, Kolkata 700026, West Bengal, India; a https://orcid.org/0000-0003-1674-5093; b https://orcid.org/0009-0003-9965-7255; c https://orcid.org/0009-0004-3968-5702; d https://orcid.org/0009-0001-3247-4257; e https://orcid.org/0009-0002-5666-3447; f https://orcid.org/0009-0002-2284-8945; g https://orcid.org/0000-0002-6325-7116; h https://orcid.org/0000-0003-0084-1283; i https://orcid.org/0000-0002-6646-7469

**Keywords:** oncology, cancer, psychology, psychiatry, psycho-oncology, LMICs, India

## Abstract

Psycho-oncology is an upcoming specialisation that encompasses the psychological and social aspects of cancer care. In an integrated psycho-oncology service, consultant psychiatrists and clinical psychologists come together with other professionals to treat comorbid psychiatric disorders of cancer patients and their caregivers. Psycho-oncologists also contribute to improving clinician-patient communication, address staff burnout and conduct translational research. The *e*cancer-Tata Medical Center Kolkata Psycho-oncology Congress, 2024 was an initiative to encourage multi-disciplinary learning in undergraduate and postgraduate medical and psychology students and early career professionals. Written feedback from 106 participants who comprised 71% (71%, 106/149) of the pre-registered delegates, showed that the overwhelming majority found the overall experience as ‘excellent’ (75/106, 70.75%) or ‘good’ (27/106, 25%). The qualitative free-text feedback expressed the desire for longer psycho-oncology conferences ‘spread over two days’, eagerness to learn about qualitative and quantitative ‘research methods’ and specific therapeutic techniques used to treat common psychiatric comorbidities in cancer patients. A video providing an overview of the event is available here: https://ecancer.org/en/video/12232-reflections-after-the-ecancer-tata-medical-center-kolkata-psycho-oncology-congress-2024. This event generated a set of educational videos on psycho-oncology that are available online here: https://ecancer.org/en/conference/1551-ecancer-tmc-kolkata-psycho-oncology-congress.

## Introduction

The majority of the current global cancer burden is in low-middle-income countries (LMICs) that have high clinical load alongside poorer health infrastructure when compared with high-income countries. In 2022, there were 0.69 million men and 0.72 million women with newly diagnosed cancer in India [1]. By 2025 the projected incidence of cancer in India is estimated to be 0.74 million men and 0.79 million women [2]. However, with improved survival, the remit of cancer care has widened beyond the control of the biological disease [3]. The emotional and social impact of cancer on the person as well as the patient’s family can be devastating, and India is no exception [4]. Psycho-oncology, an upcoming specialisation, primarily focuses on optimal management of the psychiatric comorbidities of patients with cancer, their caregivers and health care providers. The role of consultant psychiatrists is quite central in psycho-oncology. Hence undergraduate medical students would benefit from exposure to the field early in their career. Some of the crucial functions delivered by psychiatrists in cancer hospitals are: a) confirm the psychiatric diagnosis and differentiate neuropsychiatric symptoms due to underlying medical disorders (e.g., delirium) from primary psychiatric illnesses (e.g., bipolar disorder), b) rational prescription of psychiatric medications (e.g., anti-depressant and anti-psychotic medications) alongside other medications that the patient is receiving) and c) risk management (e.g., suicide risk management) and d) managing the interface of psycho-oncology services with various oncology teams. The common psychiatric diagnoses in cancer patients and their management have been published for various common cancers in India, including breast cancer [5] and head and neck cancers [6]. A recent systematic review published from Aarhas, Denmark concluded that the presence of a severe enduring mental illness is a barrier to equitable cancer care [7]. An important role of any psycho-oncology service is to ensure that patients with severe enduring mental illnesses can get optimal cancer care.

Over the past 2 decades, research in the field of psycho-oncology has studied a diverse range of topics such as the journey of patients before and during cancer treatment, social determinants of help-seeking for cancer patients, behaviors such as tobacco use in patients with cancer, end-of-life care and death rituals [8]. Research in psycho-oncology internationally has often focused on various aspects of the clinical and communication preferences of patients [9]. There has also been research on service delivery models for patients [10–12]. Psycho-oncology research in India has explored some of these topics as well. Our collaborative research with University College London has shown that cancer patients in India prefer open and honest communication with doctors [13]. Explorations of the journey of cancer patients unraveled the problems associated with geographically fragmented oncology care resulting in treatment-related migrations from one part of the country to another [4]. One of the goals of the present psycho-oncology congress was to discuss the translational aspects of research in psycho-oncology and how this could be incorporated into day-to-day clinical practice in India.

As a country, India has the highest adolescent population in the world, and one in five persons of the total Indian population is aged 10–19 years [14]. We believe any discussion on psycho-oncology should have a special session dedicated to pediatric psycho-oncology so that we do not miss out on the unmet needs of children with cancer in India [15]. Research from India has shown that children may be emotionally robust during cancer treatment but have unique information needs that are different from adults [16]. Handling young people with cancer thus requires special skills [17]. The conference had a symposium on pediatric psycho-oncology comprising three separate talks.

The complexity of the treatment plans and high clinical workload result in high rates of burnout among oncology clinicians [18]. Psycho-oncology also provides help for staff mental health in some centers in India [19]. It is not surprising that the psychological impact on the mental health of psycho-oncology staff themselves has also been acknowledged in recent years [20, 21]. The conference touched on the bidirectional impact of cancer care on staff in various presentations throughout the day.

Psycho-oncology training structures are not well defined across the world and a recent commentary on the curriculum for training psycho-oncology clinicians emphasises the need for the same [22]. Probably the best way to acquire skills to manage patients with cancer and their families is via supervised training provided by senior clinicians. Some aspects of learning could be through didactic teaching. In the absence of well-designed courses, one way to learn would be to attend short and focused training events led by experts from around the country. The *e*cancer – Tata Medical Center Kolkata Psycho-oncology Congress, 2024 was one such initiative. The academic learning as well as the motivational component of being part of a bigger group, form bonds and networks that often contribute significantly to the career growth of a professional [23]. The conference brought together a group of students, early-career clinicians and experienced practitioners to provide a foundation for learning and future networking.

*e*cancer Global Foundation has a very large repository of training videos for oncology clinicians [24]. Other than supporting face-to-face learning in conferences, e-learning modules form an important part of the work of *e*cancer Global Foundation [25]. Specialised courses catering to India on palliative medicine have been developed by *e*cancer Global Foundation [26] and much before the global pandemic, we had discussed the scope of e-learning in the field of palliative medicine in LMICs [27]. Of course, the paradigm of learning changed drastically during and following the global SARS-CoV-2 pandemic. Tata Medical Center, Kolkata has been collaborating with *e*cancer Global Foundation for several years and some of the previous conference lectures for 2019 and 2023 are also available for anyone to access [28]. The lectures of the present conference are also available online [29].

## Planning the event

**Figure 1.**
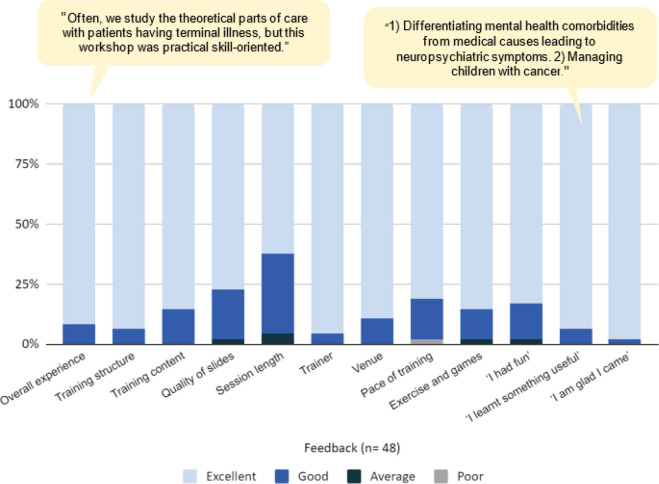
Collated feedback from participants of past psycho-oncology workshops conducted in 2023–24 at Tata Medical Center, Kolkata.

Tata Medical Center, Kolkata, has been conducting biennial psycho-oncology workshops since 2012 and the last such event was a few months before the congress in 2024. The feedback from these workshops largely guided the planning of the event. A summary of the feedback of the preparatory workshops for clinical psychologists conducted by the institution is given in Figure 1. The focus was on skill transfer from experts in the field of psycho-oncology to medical students, psychology students and young professionals at the beginning of their careers. Based on the feedback the following topics were felt to be relevant to the learning needs of the potential attendees: a) Psychological and social factors determining access to cancer care, b) managing anxiety in cancer patients, c) managing depression and suicidal risk in cancer patients and d) pediatric psycho-oncology. To make the conference more engaging and participatory, we organised a two-step pre-conference quiz competition open to everyone. The questions in the quiz covered topics of health psychology, liaison psychiatry and psycho-oncology. Additionally, original research abstracts were invited from any part of the country for selection for a poster session (Dilip Mahalanabis Poster Award Session) and an oral research presentation session (McVie-Veronessi Award Session) to be held during the conference. An anonymous feedback form was designed such that it gives the organisers an understanding of the overall acceptability of the event through quantitative ratings for various aspects of the event alongside questions that had to be answered by free text responses. The feedback was collected at the end of the congress from the participants. Video feedback on camera was also obtained from all faculty of the congress and a subgroup of consenting participants.

Dr. P Arun, Chief Executive Officer of Tata Medical Center Trust, Kolkata, acknowledged the long-standing association and collaboration between Tata Medical Center, Kolkata, and the *e*cancer Global Foundation (Video 1).

## The conference sessions

The welcome address of the conference was delivered by Dr Soumitra S Datta, organising chair for the conference. In his welcome address, Dr Datta mentioned the importance of learning about the psychological aspects of cancer care early on in the career. The welcome address included a slide on the summary statistics of the demographic information and professional background of all participants (Figure 1 and Table 1) to foster networking. The single-day psycho-oncology conference was planned such that the sessions transitioned from the basics to more complex topics and ended with original research presentations. The audience had a chance to ask questions at the end of each session. The sessions were anchored by Ms Heena Sheth and Ms Sharmili Ghosh, both Clinical Psychologists and fellows in psycho-oncology at Tata Medical Center, Kolkata, and Dr Srijan Das, post-graduate MD psychiatry trainee from Central Institute of Psychiatry, Ranchi, India.

### Session I: Help-seeking in oncology

The session on help-seeking in oncology was jointly chaired by Dr Sanjit Agrawal, Consultant Breast Oncoplastic Surgeon, and Dr Prateek Jain, Consultant Head and Neck Surgeon, both from Tata Medical Center, Kolkata. The opening lecture titled ‘Cancer Journeys: Research from India’ was by Ms Soumita Ghose where she shared the findings of collaborative research conducted by Tata Medical Center, Kolkata, and Kings College London on the access to cancer care, journeys of patients and potential disruptions to care [4]. She pointed out the patterns of intra and inter-country migration in South Asia and the difficulties faced by patients and their relatives when they travel for treatment of cancer.

The above talk was followed by a talk titled ‘Association of adult attachment with delays in accessing specialist care in women with ovarian cancer’ by Dr Soumitra S Datta, Consultant Psychiatrist at Tata Medical Center, Kolkata. This talk explored the ways adult attachment could influence help-seeking in patients citing specific examples from a research study done on treatment delay in women with ovarian cancer in Kolkata [30].

### Session II: Managing depression and suicide risk in oncology

This session was chaired by Dr Jai Ranjan Ram, Consultant Psychiatrist, Mental Health Foundation, Kolkata, and Prof Akash Mahato, Clinical Psychologist and Head of Institution, Amity Institute of Psychology and Allied Sciences, Amity University, Kolkata. Ms Bidisha Samanta, Consultant Clinical Psychologist, Rocket Health elaborated on the topic of ‘Depression in cancer’. She also emphasised the need for differentiating between overlapping physical symptoms of cancer and depression and provided strategies to prevent both the underdiagnosis and overdiagnosis of depression in cancer patients. She also covered the management of depression in cancer.

Following the above session on depression in cancer, Professor Sujit Sarkhel, IPGMER, Kolkata presented on the ‘Assessment of suicide risk in cancer patients’. He discussed the role of history-taking, assessment of the risk of completed suicide and ways to manage this risk.

### Session III: Managing anxiety in cancer

This session was chaired by Ms Aparajita Chakraborty, Assistant Professor of Clinical Psychology, Amity University, Kolkata, and Prof Sujit Sarkhel, Professor of Psychiatry at the Institute of Psychiatry, SSKM Hospital, Kolkata. During this session, Dr Arnab Mukherjee, Consultant Psychiatrist, Department of Palliative Care and Psycho-oncology, Tata Medical Center, Kolkata, discussed psychological issues in the context of hereditary cancers. His session titled ‘Psychological issues in familial cancer’ helped participants to understand the apprehensions of people who undergo tests for hereditary cancers, the role of multidisciplinary teams in familial cancer clinics, the impact of communication among family members to promote cascade testing, anxiety around positive or negative test results and role of risk reduction surgeries.

The talk on familial cancers was followed by a talk by Ms Bincy Mathew, Consultant Psycho-oncologist, Manipal Hospitals, Bengaluru titled ‘Managing ‘Fear of Recurrence’ of cancer patients’. She discussed the prevalence and associations of fear of recurrence in cancer survivors and ways to manage these symptoms through psycho-social interventions.

### Session IV: Colon cancer and mental health

In the first post-lunch session, Prof V Surendran, Head of the Department of Psycho-oncology & RCTC, Cancer Institute, Adyar, Chennai presented on ‘Colon cancer and mental health’. His presentation included psychological issues faced by people living with stoma and ways to address these.

### Session V: ecancer- ACAMH paediatric psycho-oncology symposium

The *e*cancer Global Foundation and Association of Child and Adolescent Mental Health – India Hub jointly organised the symposium on paediatric psycho-oncology as part of the main psycho-oncology congress. There were three separate talks in this symposium – all related to addressing the psychological needs of children with cancer and their families. The symposium was chaired by Dr Soheli Datta, Associate Professor of Clinical Psychology, Department of Applied Psychology, University of Calcutta, and Ms. Bidisha Samanta, Clinical Psychologist at Rocket Health, Kolkata.

Dr Soumitra S Datta, Senior Consultant, Department of Palliative Care and Psycho-oncology, TMC, Kolkata, presented on ‘Communicating with children with cancer and their families communication’. He focused on age-appropriate ways of communication and the role of the family in providing support to a child with cancer. The second talk by Ms Rhea Daruvala, Consultant Clinical Psychologist, Mazumdar Shaw Medical Centre, Bengaluru, enlightened the participants on ‘Managing sleep problems, eating difficulties and procedural anxiety in children with cancer’. She focused on behavioral and family therapy approaches to address these problems. Presentation, assessment and management of procedural anxiety in children with cancer were also discussed in this session. Dr J R Ram, Consultant Psychiatrist and Founder, Mental Health Foundation, Kolkata, spoke on ‘Managing depression and anxiety in children and young people with cancer’. He focussed on older adolescents and shared tips on establishing rapport with this very vulnerable group of patients.

### Session VI: Research award sessions

#### Dilip Mahalanabis poster award session

The Dilip Mahalanabis poster session was judged by Ms Rhea Daruvala, Consultant Psycho-oncologist, Mazumdar Shaw Medical Centre, Bengaluru, and Prof Akash Mahato, Head of the Institution, Amity Institute of Psychology and Allied Sciences, Amity University, Kolkata. The first prize winner of the poster competition was Ms Anna Jacob from Christian Medical College and Hospital, Vellore, and she presented a very well-designed poster from the field of psycho-oncology titled **‘The whole is greater than the sum of its parts’**. The second prize winner of the poster competition was Ms Anindita Mukherjee, a Clinical Psychologist from Sister Nivedita University, Kolkata, and her poster was titled **‘Quality Of Marital Life And Coping Styles Among Women With Breast Carcinoma: A Comparative Study’**.

#### McVie-Veronesi research award session

The final and last session of the conference had several presentations of original research work undertaken by the participants from different parts of the country. The participants had submitted abstracts that had gone through a competitive selection process. The session was chaired and judged by Prof Sujit Sarkhel, Professor of Psychiatry at the Institute of Psychiatry, SSKM Hospital, Kolkata, and Dr Jai Ranjan Ram, Consultant Psychiatrist and founder of Mental Health Foundation, Kolkata. The prize winners were as follows: 1) Ms Shinjini Ghosh, Department of Applied Psychology from the University of Calcutta won the first prize for her presentation titled **‘The Interplay between Cognitive Flexibility, Core-Self and the Experience of Self-Harm Among Women with Suicidal Behavior Disorder and Nonsuicidal Self-Injury: A Mixed Method Study’**. 2) Ms Jayashree K, Department of Psycho-oncology & RCTC, Cancer Institute, Chennai won the second prize for her presentation titled **‘Mapping Of Tobacco Cessation Centers In The Dental Colleges In Tamil Nadu’**. 3) Third prize was shared by Dr Rajwinder Kaur from Command Hospital, Kolkata and Ms. Dishari Choudhury from Amity University, Kolkata. Dr Kaur presented on **‘Distress The 6th Vital Sign In Cancer Care and Its Effect On Quality of Life Among Patients Availing Services From A Tertiary Care Centre In Kolkata’**. Ms Dishari Choudhury presented on **‘Self-care behavior in patients with diabetes and patients with cardiovascular disorders: Role of illness perception, perceived social support and health beliefs’**.

### Session VII: Valedictory and prize distribution ceremony

The one-day psycho-oncology congress ended by getting feedback from the participants and the prize distribution ceremony.

## Results

**Figure 2.**
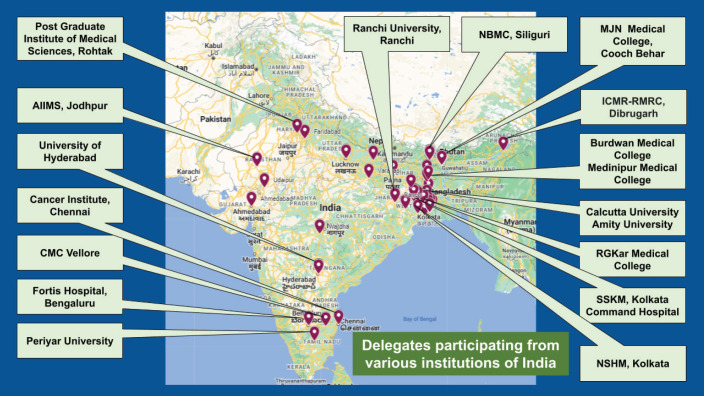
Registered delegates of the *e*cancer Tata Medical Center, Kolkata Psycho-oncology Congress 2024 from various institutions of India.

The *e*cancer-Tata Medical Center Kolkata Psycho-oncology Congress, 2024 had 149 delegates who had registered from around the country (Figure 2). Of those who registered for the conference, 106 participants (Table 1) provided written feedback. The majority of the participants were undergraduate or postgraduate students. Undergraduate medical students comprised a quarter of the participants and another quarter were those who were pursuing graduate and post-graduate courses in psychology. Another quarter were those who were psychologists or allied mental health professionals who were practicing and mostly looking after patients with cancer. There were 14 faculty members, comprising consultant psychiatrists, consultant clinical psychologists and oncology professionals who delivered the talks or chaired various academic sessions. Additionally, three key members of the organising committee (HS, SG, SD) compered the entire programme.

**Figure 3.**
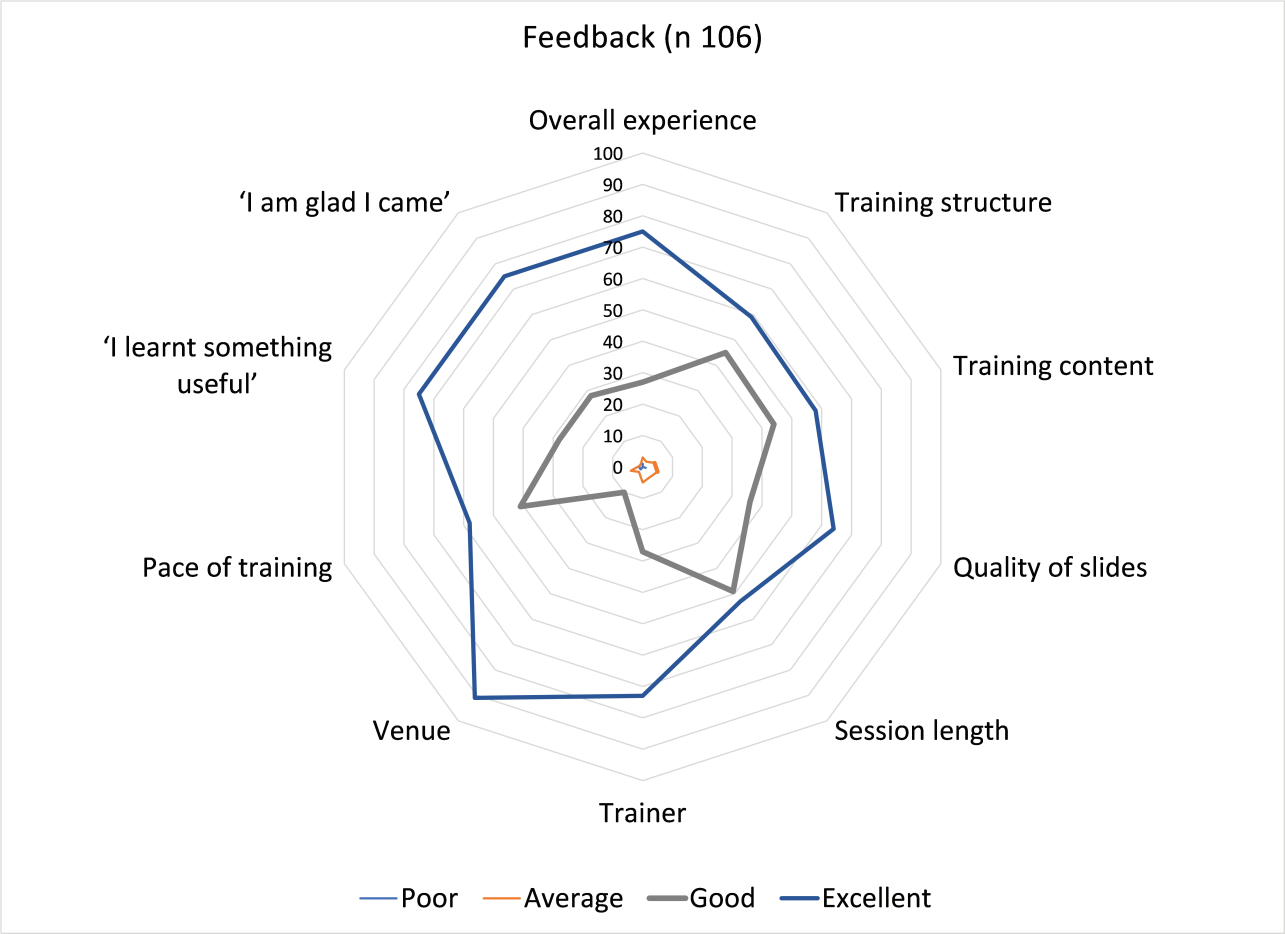
Quantitative feedback from the participants of *e*cancer TMC Kolkata Psycho-oncology Congress 2024.

As can be seen in Figure 3, the majority of the feedback from the delegates was rated ‘excellent’ followed by ‘good’ in all aspects of the quantitative ratings. In the free text part of the responses, they elaborated on specific aspects of the congress they liked (Table 2). The participants appreciated the mix of clinical and research-oriented sessions in the field of psycho-oncology.

Feedback was also collected from willing delegates in video format. They spoke about their key takeaways and reflections from the psycho-oncology congress.

## Conclusion

The *e*cancer Tata Medical Center Kolkata Psycho-oncology Congress 2024 was one of the first conferences that specifically focused on introducing psycho-oncology to clinicians in training. It had a unique blend of participants from medical backgrounds, psychology trainees and practicing psychologists, in the spirit of a truly multi-disciplinary learning event. This has been tried in other specialties [31] but has not been documented in psycho-oncology previously. The process of multi-disciplinary learning allows students from different disciplines to construct knowledge together and sows the seeds of teamworking. In a country that has increased life expectancy, improved disease outcomes and a high cancer burden, it is high time to understand the importance of psychological aspects of common medical illnesses such as cancer. This requires a paradigm shift in the way one looks at health and wellness. There is indeed no health without mental health [32].

Feedback about the event was collected from willing volunteers using multiple methods. These included anonymous written feedback, consisting of numerical ratings and open-ended responses. Additionally, video feedback was obtained from a subgroup of participants and faculty members [29]. As can be seen from the written anonymous feedback, most participants felt that the congress was valuable and rated the overall experience as ‘excellent’ (75/106, 70.75%) or ‘good’ (27/106, 25%). The anonymity of the written feedback encouraged the participants to be critical if needed. Research has shown that incorporating audio and written feedback from students enhances their motivation to improve [33]. We went one step further and engaged in anonymous written as well as video feedback collected by a peer (SD). Some of the video feedback has been incorporated in the reflections by the organising chair after the congress and is available here: https://ecancer.org/en/video/12232-reflections-after-the-ecancer-tata-medical-center-kolkata-psycho-oncology-congress-2024 (Video 2).

Several participants requested follow-up events that are participatory skill-building sessions and this is already being planned by the organisers. These events have to be small group teachings that follow a workshop format. In future congresses, we also will try to incorporate in-person polls through platforms like PollEV [34] that may engage the learners more actively. More research is needed on ways to develop better methods of teaching in multidisciplinary teams to break up the barriers of interactions, promote cohesive teamwork and make cancer care truly patient-centered. We have also developed a website that supports the learning and development of undergraduate and postgraduate trainees and health care professionals that can be accessed here: https://artofmedicine.in/.

## Conflicts of interest

The authors declared they have no competing interests.

## Figures and Tables

**Figure 4.**
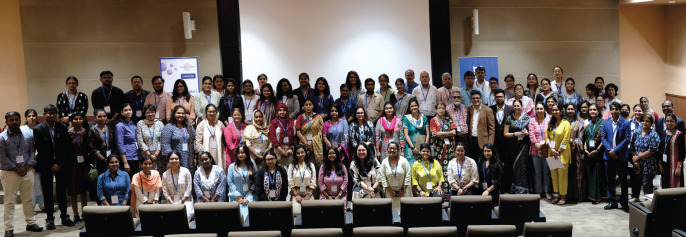
Faculty and delegates of the *e*cancer-Psycho-oncology Congress 2024.

**Video 1.**
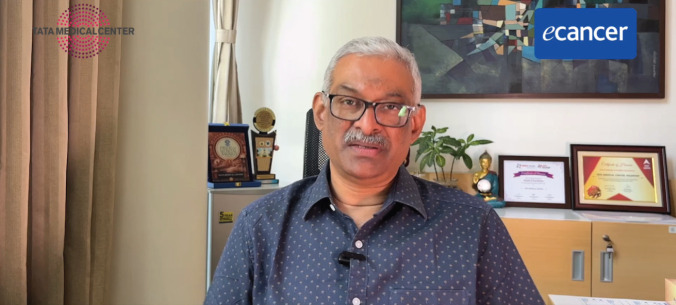
Dr P Arun, Chief Executive Officer of Tata Medical Center Trust, Kolkata, spoke on the long-standing partnership between Tata Medical Center, Kolkata and *e*cancer Global Foundation (https://ecancer.org/en/video/12231-the-long-standing-partnership-between-tata-medical-center-kolkata-and-ecancer-global-foundation).

**Video 2.**
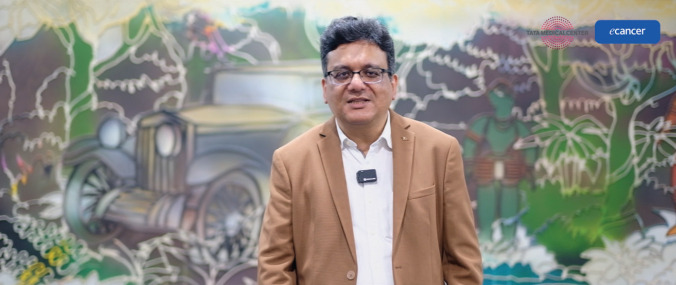
Reflections after the *e*cancer Tata Medical Center, Kolkata Psycho-oncology congress 2024 by the participants and Dr Soumitra S Datta, the congress chair (https://ecancer.org/en/video/12232-reflections-after-the-ecancer-tata-medical-center-kolkata-psycho-oncology-congress-2024).

**Table 1.** Demographic and educational background of delegates who provided written feedback for the congress.

Variable	*n* = 106	Frequency	Percentage
Age range	16–21	17	16%
	22–30	61	58%
	31–40	15	14%
	41–50	10	9%
	51–60	3	3%
Gender	Male	26	24%
	Female	76	72%
	Preferred not to mention	4	4%
Specialty	MBBS students	29	27%
	Undergraduate and post graduate psychology students	17	16%
	Psychologists (Counseling, psycho-oncologist, clinical psychologist)	23	22%
	Qualified doctors	13	12%
	PhD Research Scholar	2	2%
	Others (NGO, teachers, managers etc.)	22	21%

**Table 2.** Qualitative feedback from the participants of *e*cancer-Tata Medical Center Kolkata Psycho-oncology Congress 2024.

What do you like about the conference?	How do you think the conference could be made more interesting and improved?	Outline three things that you have learned in this conference	Do you have any suggestions for a new session?
*‘1) Training structure* *2) Training content* *3) Slide presentations (High-quality slides)* *4) Venue & refreshment* *5) Session length* *6) Excellent knowledge exchange* *7) Very good trainers’*	*‘1) The slides should be made such that they are visible clearly at the back’*	*‘1) Mental health issues faced by cancer survivors* *2) Detecting suicidal ideations in cancer patients* *3) Social stigma related to various cancers like breast cancer, ovarian cancer and colorectal cancer’*	*‘It would be great if this could be spread across 2 days to allow for more enriching discussions on topics explored’.*
*‘I liked this workshop because I think psycho-oncology is now recognised as an essential part of quality cancer care. Psycho-oncology addresses the emotional aspects of cancer patients and their family members’.*	*‘1) Management of some psychological symptoms was covered briefly. It'll be great if this topic can be dealt with in more detail.* *2) It can also be interactive (e.g. small skills training/role play of topics etc.)’*	*‘1) The role of attachment in seeking help 2) The various factors that can act as facilitators and barriers for help seeking 3) The psychological issues associated with positive and negative test results in familial cancer’*	*‘I want to learn about behavioral therapy as applicable to cancer patients, management of suicide risk, and ways to counsel family members to support the patient’.*
*‘I liked the way we were taught to address mental health issues in cancer patients’.*	*‘It would better if there are more group discussions and hands-on exercises’.*	*‘1) Fear of cancer recurrence 2) Modifiable & nonmodifiable risk factors of cancer 3) depression & suicide risk in cancer’*	*‘You can organise mini quizzes in between sessions so that the audience can be actively involved throughout the day’.*
*‘I am an undergraduate student. I learned how to present a research paper. This was very useful to me’.*	*‘The conference can also be a multi-day event’.*	*‘1) Learnt about qualitative & quantitative research papers 2) Learnt how to present papers 3) Mental health stigmas associated with having cancer’.*	*‘I want to learn how to cope with managing patients with progressive disease receiving end of life care’*

## References

[1] World Health Organization: International Agency for Research on Cancer (2022). Cancer Today.

[2] World Health Organization: International Agency for Research on Cancer (2022). Cancer Tomorrow.

[3] Johnson S, Adams C (2023). Invited editorial: why all countries should include psycho-oncology in their cancer response. Psychooncology.

[4] Datta SS, Ghose S, Ghosh M (2022). Journeys: understanding access, affordability and disruptions to cancer care in India. Ecancermedicalscience.

[5] Mukherjee A, Samanta B, Sharma V (2024). When do patients with breast cancer seek help from psycho-oncology services? A 3-year retrospective study from India. Indian J Med Paediatr Oncol.

[6] Mukherjee A, Bhowmick C, Chattopadhyay S (2022). Preoperative risk factors associated with peri-operative psychiatric diagnosis in oral cancer patients. Ecancermedicalscience.

[7] Bentson TM, Fløe LE, Bruun JM (2023). Barriers in cancer trajectories of patients with pre-existing severe mental disorders-a systematic review. Psychooncology.

[8] Conti I, Davidson M, Cutress RI (2024). Global trends in psycho-oncology research investments 2016–2020: a content analysis. Psychooncology.

[9] Westendorp J, Geerse OP, Lee ML (2023). Harmful communication behaviors in cancer care: a systematic review of patients and family caregivers perspectives. Psychooncology.

[10] King A (2016). The next challenge for psycho-oncology in the UK: targeting service quality and outcomes. Future Oncol.

[11] Mukherjee A, Chatterjee M, Chattopadhyay S (2021). Psycho-oncology service provisions for hospitalised cancer patients before and during the COVID-19 pandemic in an oncology centre in eastern India. Ecancermedicalscience.

[12] Hui D, Hoge G, Bruera E (2021). Models of supportive care in oncology. Curr Opin Oncol.

[13] Datta SS, Tripathi L, Varghese R (2017). Pivotal role of families in doctor-patient communication in oncology: a qualitative study of patients, their relatives and cancer clinicians. Eur J Cancer Care (Engl).

[14] Children in India. https://www.unicef.org/india/children-in-india.

[15] Datta SS (2023). Children with cancer: are we healing the body & missing the mind?. Indian J Med Res.

[16] Chaudhuri T, Nandakumar D, Datta SS (2022). Information-sharing experiences of professionals looking after children with cancer: a qualitative exploration from a specialist paediatric oncology unit in India. Ecancermedicalscience.

[17] Datta SS, Saha T, Ojha A (2019). What do you need to learn in paediatric psycho-oncology?. Ecancermedicalscience.

[18] Eelen S, Bauwens S, Baillon C (2014). The prevalence of burnout among oncology professionals: oncologists are at risk of developing burnout: burnout in oncology: oncologists at risk. Psychooncology.

[19] Daruvala R, Ghosh M, Fratazzi F (2019). Emotional exhaustion in cancer clinicians: a mixed methods exploration. Indian J Med Paediatr Oncol.

[20] Engler‐Gross A, Goldzweig G, Hasson‐Ohayon I (2020). Grief over patients, compassion fatigue, and the role of social acknowledgment among psycho‐oncologists. Psycho-Oncology.

[21] Andersen BL, Dorfman CS, Conley CC (2021). Achieving oncology mental health providers’ usage of an empirically supported treatment: lessons learned. Psychooncology.

[22] Grassi L, Watson M (2024). Core-curriculum in psycho-oncology: towards the certification of the psycho-oncologist profession. Psychooncology.

[23] Blanchard RD, Engle DL, Howley LD (2016). From the coliseum to the convention centre: a reflection on the current state of medical education conferences and conference-goers. Med Educ.

[24] *e*cancer Global Foundation (2024). Video latest – *e*cancer. http://ecancer.org/en/video.

[25] elearning latest – *e*cancer. http://ecancer.org/en/elearning.

[26] Palliative care e-learning course for healthcare professionals in India – *e*cancer. https://ecancer.org/en/elearning/module/196-basic-principles-of-palliative-care?next_uri=/en/elearning/module/196-basic-principles-of-palliative-care/learn.

[27] Datta SS, Agrawal S (2017). Can e-learning help you to connect compassionately? Commentary on a palliative care e-learning resource for India. Ecancermedicalscience.

[28] 2nd *e*cancer – TMC Kolkata Oncology Congress - *e*cancer. http://ecancer.org/en/conference/1505-2nd-ecancer-tmc-kolkata-oncology-congress.

[29] *e*cancer - TMC Kolkata Psycho-oncology Congress - *e*cancer. http://ecancer.org/en/conference/1551-ecancer-tmc-kolkata-psycho-oncology-congress.

[30] Datta SS, Fraser L, Burnell M (2022). Association of adult attachment with delays in accessing specialist care in women with ovarian cancer. J Psychosoc Oncol.

[31] Stephens M, Robinson L, McGrath D (2013). Extending inter-professional learning through the use of a multi-disciplinary Wiki. Nurse Educ Pract.

[32] Prince M, Patel V, Saxena S (2007). No health without mental health. Lancet.

[33] Kirwan A, Raftery S, Gormley C (2023). Sounds good to me: a qualitative study to explore the use of audio to potentiate the student feedback experience. J Prof Nurs.

[34] Engage your audience. https://www.polleverywhere.com/.

